# Minimal Invasive Percutaneous Osteosynthesis for Elderly Valgus Impacted Proximal Humeral Fractures with the PHILOS

**DOI:** 10.1155/2015/971216

**Published:** 2015-11-26

**Authors:** Hang Chen, Xiaochuan Hu, Haochen Tang, Guoyong Yang, Ming Xiang

**Affiliations:** Sichuan Orthopaedic Hospital, No. 132 Yihuan Road, Chengdu, Sichuan 610041, China

## Abstract

There is a growing concern about elderly valgus impacted proximal humeral fractures. The aim of this study was to evaluate the treatment and clinical outcomes following minimal invasive percutaneous plate osteosynthesis (MIPPO) with the proximal humeral internal locking system (PHILOS) for the treatment of elderly valgus impacted proximal humeral fracture. Between May 2008 and May 2012, 27 patients (average age 67.3, range 61–74) with valgus impacted proximal humeral fractures were enrolled in the study. The patients were treated with MIPPO using PHILOS-plate through the anterolateral delta-splitting approach. Rehabilitation exercises were done gradually. The NEER score and Constant-Murley score were used to evaluate shoulder function. All the patients were followed up by routine radiological imaging and clinical examination. There were 15 cases of II-part greater tuberosity fractures, 10 cases of III-part greater tuberosity fractures, and 2 cases of IV-part fractures according to the NEER classification. The surgery was successful in all patients with an average follow-up of 20.8 (range: 11–34) months. The fractures united in an average of 7.2 (6–14) weeks without implant loosening. According to NEER score, there were 17 excellent, 7 satisfactory, 2 unsatisfactory, and 1 poor. The mean Constant-Murley score was 89.4 ± 4.35. No complication including axillary nerve damage, postoperative nerve or vessel damage, infections, DVT, or death was observed. In conclusion, the MIPPO technique with the PHILOS through the anterolateral delta-splitting approach seems to be a safe and easy treatment for elderly valgus impacted proximal humeral fractures. A case-control study and longer follow-up time are needed.

## 1. Introduction

The proximal humeral fracture is a common fracture of the upper extremity [1]. Valgus impacted proximal humeral fractures present several problems, such as complex anatomy, risk of avascular necrosis [2], and minimal bone stock, which must be considered in order to achieve satisfactory treatment results. It is reported that nonoperative treatment has been proved successful in many cases, especially in the patients with undisplaced and minimally displaced fractures [3]. The traditional anterior deltopectoral approach has been most commonly used for plating of the proximal humerus. However, this approach requires extensive soft tissue dissection and may impair the anterior circumflex humeral artery; the exposure of the plating zone is different [4]. Alternatively, the anterolateral deltoid-splitting approach minimizes soft tissue dissection and has the merits of easy access and excellent visualization of the greater tuberosity and the plating area [5]. The anterolateral deltoid splitting simplifies posterior positioning of the plate to allow for better capture of the greater tuberosity. It provides easier access to the infraspinatus insertion for application of stay sutures. Another benefit is indirect reduction by ligamentotaxis and by reduction to the plate in a valgus displaced fracture configuration [6].

The proximal humerus internal locking system provides angular stability and has been used for operative management of proximal humeral fractures for several years. The system has the potential for enhanced stability of bone-plate structure that could allow early functional exercises. Additionally, it can be inserted using a MIPPO approach without additional damage [5, 7]. This combination offers a good option for the treatment of proximal humeral fractures, better functional outcomes, and shorter hospital stays.

Due to increase in ageing population and the osteoporosis, osteoporotic fractures in the elderly have been the focus of recent studies [8]. Additionally, proximal humeral fracture is the third most common fracture in elderly people [1]. Therefore, the aim of this study was to evaluate the treatment and clinical outcomes following MIPPO with PHILOS-plate for the treatment of valgus impacted proximal humeral fracture in elderly people. In this study, we presented our experience of 27 cases that had this surgery.

## 2. Materials and Methods

This study was approved by the ethics committee of Sichuan Orthopaedic Hospital and signed informed consent forms were obtained from all patients. Patients sustaining a valgus impacted proximal humeral fracture between May 2008 and May 2012 were enrolled in the study. The patients were treated with MIPPO using PHILOS-plate. Inclusion criteria were a valgus impacted proximal humerus fracture as diagnosed with imaging and suitable bone for surgery [6]. Patients with comminuted head fracture, primary neurovascular damage, or obesity were excluded.

The 27 cases (Table 1) were diagnosed as valgus impacted proximal humeral fractures according to the NEER classification. The average diagnostic T value of bone mineral density was −1.8 (range: −0.8 to −3.1). According to the intraoperative bone loss, the patients were treated with allograft strut bone graft.

**Figure 1:**
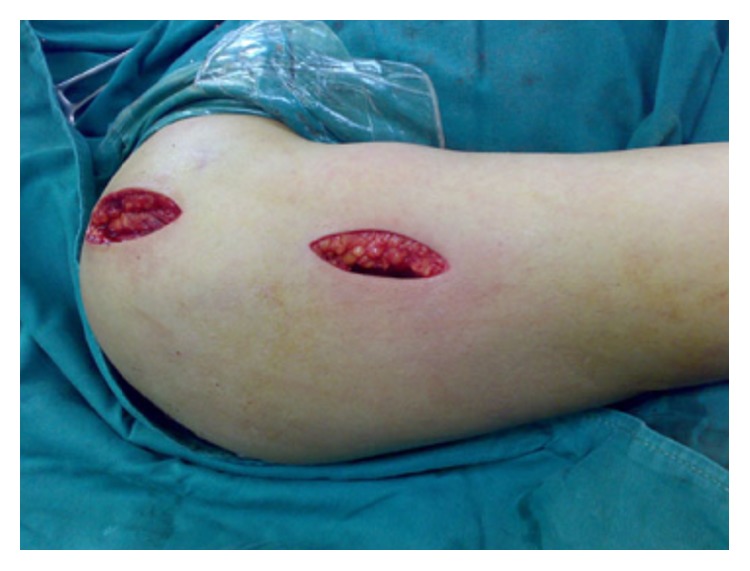
MIPPO with anterolateral delta-split approach in a patient with II-part greater tuberosity fracture: the skin incision at the anterolateral tip of the acromion.

**Figure 2:**
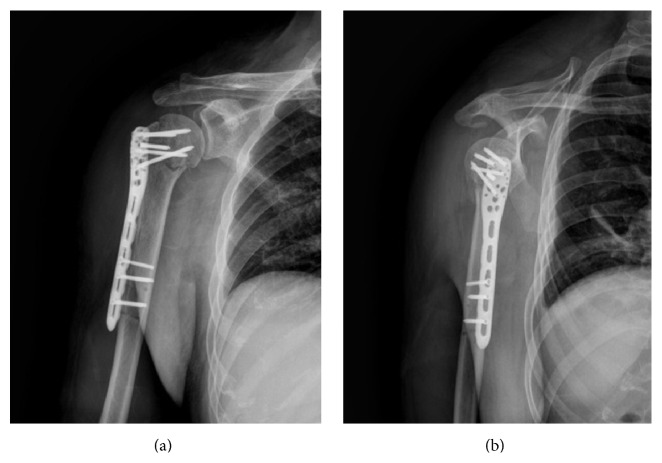
(a) Anteroposterior view and (b) lateral view of plate fixation and reduction in a patient with II-part greater tuberosity fracture.

Imaging with X-ray and CT was performed preoperatively to evaluate the fracture type. Patients were in a beach chair position under general anesthesia. A skin incision was made at the anterolateral tip of the acromion extending approximately 5 cm distally (Figure 1). Triangular muscle fibers were tagged with suture in the distal incision. The pectoral fascia was opened to expose the humeral head and the tuberosity of the humerus. The tendon on the surface of the greater tuberosity was tagged with nonabsorbable suture. The greater tuberosity was flipped to reveal the surface of humeral head. The fracture was then reduced and grafted if needed. The greater tuberosity and humeral head were pinned provisionally with one or two Kirschner wires. If the lesser tuberosity was injured, it was pinned as well. The reduction was confirmed with fluoroscopy. A 5-hole PHILOS-plate was used. A 3 cm incision was made longitudinally underneath the proximal incision to expose the anterolateral humerus. The plate was inserted from proximal to distal (Figure 2). Fluoroscopy was used to adjust the plate to an appropriate height. The plate was fixed with 4 or 5 screws proximally and 2 or 3 screws distally (Figure 2). If the lesser tuberosity was injured, it was fixed directly to humeral head with a cortical bone screw from proximal to distal. The incision was closed.

Postoperative rehab consisted of elbow flexion to 90° and external rotation to 0° for 3-4 weeks in order to reduce tension on the greater tuberosity and promote healing. Two days postoperatively, the patient started passive motion exercise with shoulder forward flexion to 45°. One week postoperatively, the patients were encouraged to start passive mobilization of the shoulder with forward flexion to 60° and external rotation to 10°. Three to four weeks after the surgery, passive mobilization of the shoulder with forward flexion to 90° and external rotation to 30° was begun. Depending on healing progression on X-ray, patients started active exercise with forward flexion and external rotation at 5-6 weeks postoperatively. Seven to eight weeks postoperatively, patients started active shoulder joint internal rotation. Three months postoperatively, resistance exercise was encouraged.

Outcomes were evaluated by NEER shoulder joint function score and Constant-Murley shoulder grading system. The data were expressed as mean ± SD.

All patients were followed up with routine radiological imaging and clinical examination every 2-3 weeks within the first four months. Further imaging and examination were performed depending on the fracture healing.

## 3. Results

Twenty-seven patients met the inclusion criteria and were enrolled in the study, including 15 cases of II-part greater tuberosity fractures, 10 cases of III-part greater tuberosity fractures, and 2 cases of IV-part fractures according to the NEER classification (Table 1). Fifty-six patients were excluded. The surgery was successful in all patients with an average surgery time of 52 min (range: 40–70 min). The intraoperative blood loss was 70 mL on average (range: 50–100 mL). Seven cases were treated with allograft due to intraoperative bone loss. Average follow-up was 20.8 months (range: 11–34).

**Figure 3:**
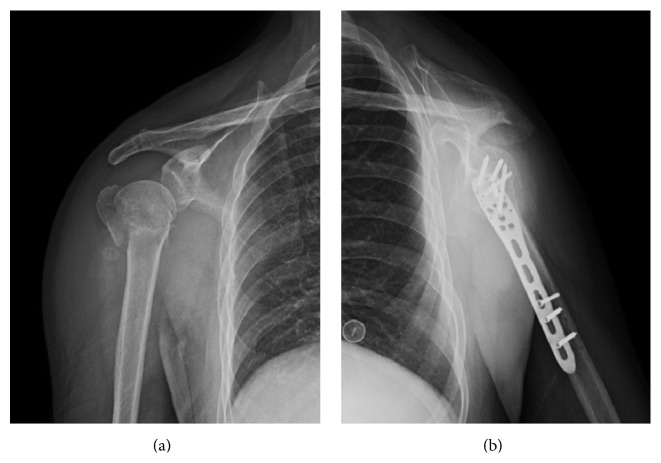
Preoperative (a) and postoperative (b) radiograph (lateral view) of proximal humeral fractures in a patient with II-part greater tuberosity fracture.

All fractures healed satisfactorily without internal fixation failure or reduction loss, except for one case. The fractures united in an average of 7.2 (6 to 14) weeks without implant loosening (Figure 3). One case with a IV-part fracture presented with cystic density decrease in the humeral head and screw cutout 6 months postoperatively. The implant was removed 11 months postoperatively. Joint wear was observed under glenohumeral arthroscopy and joint pain was relieved after debridement.

**Figure 4:**
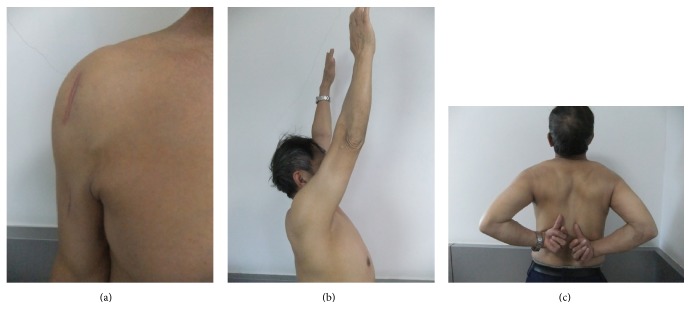
(a) The incision healed during the follow-up in a patient with II-part greater tuberosity fracture. ((b) and (c)) After removal of the implant, the patient has good function.

In terms of function, there were 17 excellent, 7 satisfactory, 2 unsatisfactory, and 1 poor outcome according to the NEER score. The mean Constant-Murley score was 89.4 ± 4.35 points (Figure 4). The final Constant-Murley score was very high for this aged population. We attributed it to the following reasons. First, the patients were valgus impacted fractures and their scores were relatively high. Second, the injuries were relatively minor with a normal rotator cuff, which contributed to a good result. The average range of motion and pain score were 20.92 ± 3.46 and 31.25 ± 2.26, respectively, according to the NEER score. No other complication including axillary nerve damage, postoperative nerve or vessel damage, infections, DVT, or death was observed.

## 4. Discussion

Today the most used approach for proximal humerus fracture is the anterior deltopectoral approach. It provides adequate exposure for internal fixation but requires extensive soft tissue dissection and muscle retraction to gain adequate exposure of the lateral and posterolateral aspect of the humeral head. It is very difficult to reduce and fix posterior parts of the greater tuberosity. To keep the deltoid muscle away from the fracture zone, retractors have to be positioned behind the humerus itself. The deforming force at the fracture site results in an anterior angulation which may complicate the reduction [4, 9].

In contrast, the anterolateral deltoid-splitting approach is placed directly over the main fracture zone and offers better access to the greater tuberosity. No large retractors are necessary to keep the deltoid muscle away from the fracture. This method requires less soft-tissue dissection and restricts fracture hematoma and damage to the blood supply to the bone fragments [10, 11]. Therefore, in our clinical practice, the reduction of the main fragments (greater tuberosity to head, head to shaft) can be achieved easily. In the present study, the 27 cases with valgus impacted fractures were treated with this approach and obtained satisfactory results.

The risk factor of the delta-splitting approach is the axillary nerve damage [12]. In contrast, axillary nerve injury is rarely seen with the deltopectoral approach. The location of the nerve can be easily predicted. It crosses the lateral aspect of the proximal humerus in a range of 6.1 ± 0.7 cm below the cranial tip of the humerus [13]. Moreover, it is reported that if the locking screws are limited to superior and inferior holes, the placement of a locking proximal humerus plate via a minimally invasive lateral trans-deltoid approach is safe [14]. We fixed the plate to the humeral head with the four most proximal screws which could be placed through this short approach. Therefore, the axillary nerve stayed below the screw position. In the present study, no axillary nerve damage was observed. Similarly, in the study by Acklin et al., there was one case (1/29, 3.4%) with axillary nerve injury after osteosynthesis with MIPPO through the anterolateral delta-splitting approach [15].

This approach may minimize the soft tissue damage and improve bone healing, leading to less infection and postoperative complications [6, 16]. In the present study, the fractures united in an average of 7.2 (6 to 14) weeks without implant loosening. Compared with the previous reports [4, 15, 17], this fracture union time was much shorter. No complication including axillary nerve damage, postoperative nerve or vessel damage, infections, DVT, or death was observed [4, 15, 17, 18]. However, it is important to note that this approach is technically demanding, since the surgical exposure and fracture reduction are limited. Another disadvantage of this technique is potential implant impingement, which may lead to limited forward flexion and sometimes require a secondary operation [6]. To solve this problem, we suggest that low profile anatomical proximal humerus plates should be used.

In the present cases with a similar injury mechanism, the patients sustained greater tuberosity fracture due to the axial load. In elderly patients, these injuries are of low energy and have a relatively complete soft tissue hinge, without obvious shift inside the bone cortex [11]. In such cases, satisfactory treatment with MIPPO can be achieved. However, when fractures involve serious soft tissue hinge damage, it is hard to achieve satisfactory reduction. Therefore, it is necessary to fully understand the injury mechanism and fracture type in order to select the appropriate surgical procedure and achieve the ideal therapeutic effect.

In our study, with an average postoperative follow-up of 20.8 months, satisfactory outcomes were achieved which was demonstrated by the NEER score, Constant-Murley score, and radiological imaging. Limitations of this study include absence of prospective data and short follow-up time. In conclusion, the MIPPO technique with the PHILOS through the anterolateral delta-splitting approach seems to be a safe and easy treatment for elderly valgus impacted proximal humeral fractures. Further research into this topic is needed.

## Figures and Tables

**Table 1:** Demographics of the patients.

Characteristic		Value
Gender	Male/female	17/10

Age	Average	67.3 (61–74)

Mechanism	Traffic accident	9
	Fall	14
	Sports	4

NEER type	II	15
	III	10
	IV	2

Follow-up (month)	Average	20.8 (11–134)

NEER score	Average	88.30 ± 7.26

Constant-Murley score	Average	89.4 ± 4.35

## References

[1] Baron J. A., Barrett J. A., Karagas M. R. (1996). The epidemiology of peripheral fractures. *Bone*.

[2] Helmy N., Hintermann B. (2006). New trends in the treatment of proximal humerus fractures. *Clinical Orthopaedics and Related Research*.

[3] Olerud P., Ahrengart L., Ponzer S., Saving J., Tidermark J. (2011). Hemiarthroplasty versus nonoperative treatment of displaced 4-part proximal humeral fractures in elderly patients: a randomized controlled trial. *Journal of Shoulder and Elbow Surgery*.

[4] Zhou Z.-B., Gao Y.-S., Tang M.-J., Sun Y.-Q., Zhang C.-Q. (2012). Minimally invasive percutaneous osteosynthesis for proximal humeral shaft fractures with the PHILOS through the deltopectoral approach. *International Orthopaedics*.

[5] Fjalestad T., Hole M. Ø., Blücher J., Hovden I. A. H., Stiris M. G., Strømsøe K. (2010). Rotator cuff tears in proximal humeral fractures: an MRI cohort study in 76 patients. *Archives of Orthopaedic and Trauma Surgery*.

[6] Acklin Y. P., Sommer C. (2012). Plate fixation of proximal humerus fractures using the minimally invasive anterolateral delta split approach. *Operative Orthopadie und Traumatologie*.

[7] Clement H., Pichler W., Tesch N. P., Heidari N., Grechenig W. (2010). Anatomical basis of the risk of radial nerve injury related to the technique of external fixation applied to the distal humerus. *Surgical and Radiologic Anatomy*.

[8] Cummings S. R., Melton L. J. (2002). Osteoporosis I: epidemiology and outcomes of osteoporotic fractures. *The Lancet*.

[9] Magovern B., Ramsey M. L. (2008). Percutaneous fixation of proximal humerus fractures. *Orthopedic Clinics of North America*.

[10] Gardner M. J., Weil Y., Barker J. U., Kelly B. T., Helfet D. L., Lorich D. G. (2007). The importance of medial support in locked plating of proximal humerus fractures. *Journal of Orthopaedic Trauma*.

[11] Kristiansen B., Andersen U. L. S., Olsen C. A., Varmarken J.-E. (1988). The Neer classification of fractures of the proximal humerus—an assessment of interobserver variation. *Skeletal Radiology*.

[12] Brunner A., Thormann S., Babst R. (2012). Minimally invasive percutaneous plating of proximal humeral shaft fractures with the Proximal Humerus Internal Locking System (PHILOS). *Journal of Shoulder and Elbow Surgery*.

[13] Duparc F., Muller J.-M., Fréger P. (2001). Arterial blood supply of the proximal humeral epiphysis. *Surgical and Radiologic Anatomy*.

[14] Smith J., Berry G., Laflamme Y., Blain-Pare E., Reindl R., Harvey E. (2007). Percutaneous insertion of a proximal humeral locking plate: an anatomic study. *Injury*.

[15] Acklin Y. P., Jenni R., Walliser M., Sommer C. (2009). Minimal invasive PHILOS-plate osteosynthesis in proximal humeral fractures. *European Journal of Trauma and Emergency Surgery*.

[16] Gardner M. J., Voos J. E., Wanich T., Helfet D. L., Lorich D. G. (2006). Vascular implications of minimally invasive plating of proximal humerus fractures. *Journal of Orthopaedic Trauma*.

[17] Jiang R., Luo C.-F., Zeng B.-F., Mei G.-H. (2007). Minimally invasive plating for complex humeral shaft fractures. *Archives of Orthopaedic and Trauma Surgery*.

[18] Panagopoulos A. M., Dimakopoulos P., Tyllianakis M. (2004). Valgus impacted proximal humeral fractures and their blood supply after transosseous suturing. *International Orthopaedics*.

